# Mouse Models of Mutations and Variations in Autism Spectrum Disorder-Associated Genes: Mice Expressing *Caps2/Cadps2* Copy Number and Alternative Splicing Variants

**DOI:** 10.3390/ijerph10126335

**Published:** 2013-11-27

**Authors:** Tetsushi Sadakata, Yo Shinoda, Akira Sato, Hirotoshi Iguchi, Chiaki Ishii, Makoto Matsuo, Ryosuke Yamaga, Teiichi Furuichi

**Affiliations:** 1 Advanced Scientific Research Leaders Development Unit, Gunma University, Maebashi, Gunma 371-8511, Japan; E-Mail: sadakata-1024@umin.ac.jp; 2 JST-CREST, Kawaguchi, Saitama 332-0012, Japan; 3 Department of Applied Biological Science, Tokyo University of Science, Noda, Chiba 278-8510, Japan; E-Mails: yshinoda@rs.tus.ac.jp (Y.S.); asato@rs.tus.ac.jp (A.S.); python357@hotmail.co.jp (H.I.); atmo_sphere@hotmail.co.jp (C.I.); makoto03010301@gmail.com (M.M.); ryohd@vm01.vaio.ne.jp (R.Y.)

**Keywords:** CAPS2/CADPS2, dense-core vesicle, BDNF, GABAergic interneurons, CNV, ASD mouse model, social interaction, anxiety, maternal behavior

## Abstract

Autism spectrum disorder (ASD) is a neurodevelopmental disorder characterized by disturbances in interpersonal relationships and behavior. Although the prevalence of autism is high, effective treatments have not yet been identified. Recently, genome-wide association studies have identified many mutations or variations associated with ASD risk on many chromosome loci and genes. Identification of the biological roles of these mutations or variations is necessary to identify the mechanisms underlying ASD pathogenesis and to develop clinical treatments. At present, mice harboring genetic modifications of ASD-associated gene candidates are the best animal models to analyze hereditary factors involved in autism. In this report, the biological significance of ASD-associated genes is discussed by examining the phenotypes of mouse models with ASD-associated mutations or variations in mouse homologs, with a focus on mice harboring genetic modifications of the *Caps2*/*Cadps2* (Ca^2+^-dependent activator protein for secretion 2) gene.

## 1. Introduction

Autism is a neurodevelopmental disorder that occurs more frequently among male than female individuals, with a male to female ratio of 4:1, and is usually diagnosed by 3 years of age. It is characterized by qualitative impairments in social interactions, verbal and non-verbal communication skills, limited interest and repetitive, stereotyped behavior [1,2]. Autism spectrum disorder (ASD) encompasses classical autism, Asperger syndrome (which lacks impairments in language and cognitive function), and Pervasive Developmental disorder-Not otherwise Specified (PDD-NOS), in which the extent of the disorder is not as severe with a delayed disease onset. The fifth edition of the Diagnostic and Statistical Manual of Mental Disorders (DSM-5), describing the global diagnostic standards established by the American Psychiatric Association, has been published [3]. Within DSM-5, diagnoses classified as autism, Asperger syndrome, PDD-NOS, and childhood disintegrative disorder, are unified and generalized as ASD. Furthermore, three characteristic symptoms that were the traditional diagnostic standards have been incorporated into two core symptoms, “social communication” and “limited interest and repetitive behavior.” 

ASD prevalence is high with a rate of 1–2 cases per 100 people, and patients with ASD do not fully recover [1,2], leaving a heavy burden on family members. Consequently, ASD is emerging as a serious problem worldwide. The involvement of heredity factors in ASD is strongly indicated, and environmental and epigenetic factors are also thought to be associated with disease risk [4,5,6,7]. However, the underlying molecular mechanisms remain unknown. Various mutations or variations related to ASD have been identified through a genome-wide association study (GWAS) [4,5]. Moreover, recent exome sequencing studies have identified *de novo* mutations in various candidate genes [8,9,10,11], and recurrent disruptive mutations of certain genes [12]. Thorough understanding of the basic biology of ASD is required to develop effective diagnostic and treatment methods [2]. Therefore, it is necessary to conduct basic research on heredity and environmental factors by developing animal models in which ASD-associated genes have been modified. 

## 2. Risk Factors for ASD

### 2.1. Heredity Factors

The involvement of heredity factors in ASD is strongly suggested by high concordance rates in monozygotic (70%–90%) and dizygotic (0%–10%) twins, and a high risk rate for developing ASD among siblings of a proband (25 times higher than normal) [13]. Various mutations or variations associated with ASD have been identified by studies, including GWAS. Among these mutations or variations, rare single nucleotide polymorphisms (SNPs) [14] and *de novo* copy number variations (CNVs), in which a single allele of a pair has a deletion or duplication, have been identified [15,16,17,18,19] (Table 1). It has been reported that *de novo* CNV contribute to ASD vulnerability in 5%–10% of idiopathic ASD onset cases [6]. Association between a father’s age and mutation rate has also been suggested [20].

**Table 1:** ASD-associated genes and their functions.

Gene symbol (Cytoband)	Molecular function, property	Gene symbol (Cytoband)	Molecular function, property
DISC1 (1q42.1)	LIS1/NUDE1/14-3-3ε, PDE4B, GSK3/β−catenin pathway	PTPN11 (12q24)	Noonan syndrome, protein Tyr phospholyase
NRXN1 (2p16.3)	Cell adhesion, synapse assembly, interact with NLGL1	CHD8 (14q11.2)	DNA helicase, transcription repressor, β−catenin binding
SLC25A12 (2q24)	Mitochondrial Asp/Glu transport	CYFIP1 (15q11)	Interact with FMR1 and WAVE
TBR1 (2q24)	T-box brain protein 1, transcriptional regulation	UBE3A (15q11.2)	Ubiquitin ligase, Angelman syndrome
OXTR (3p25)	Oxytocin receptor (Gq-PLC-coupled)	GABRB3 (15q11.2-q12)	GABA(A) receptor beta-3 subunit
CNTN4 (3p26-p25)	Immunoglobulin superfamily cell adhesion, axon connection	PRKCB (16p11.2)	Ca^2+^-activated protein kinase C-β
SLC9A9 (3q24)	Sodium/proton exchanger, late recycling endosome	CACNA1H (16p13.3)	Voltage dependent Ca^2+^ channel alpha 1H subunit
C3orf58 (3q24)	Uncharacterized, activity-dependent expression	TSC2 (16p13.3)	Tuberous sclerosis protein, regulate Rheb GTPase & mTOR
TBL1XR1 (3q26.32)	Transducin (beta)-like 1 X-linked receptor 1,	A2BP1/FOX1 (16p13.3)	RNA binding, RNA transport, splicing regulator
JAKMIP1 (4p16.1)	Interact with Janus kinase and microtubule	CREBBP (16p13.3)	Transcriptional coactivator, Rubinstein-Taybi syndrome
CDH10 (5p14.1)	Cell adhesion (type II classical cadherin)	RAI1 (17p11.2)	Transcription factor, Smith-Magenis syndrome
CDH9 (5p14.1)	Cell adhesion (type II classical cadherin)	NF1 (17q11.2)	Neurofibromatosis type I, negative regulator of Ras
SEMA5A (5p15.2)	Axon guidance molecule, neural development	SLC6A4 (17q11.1-q12)	Serotonin plasma membrane transporter
NIPBL (5p13.2)	Sister chromatid cohesion, Cornelia de Lange syndrome	ITGB3 (17q21.32)	Cell adhesion & cell surface signaling, integrin β-chain
NSD1 (5q35.3)	Transcription coregulator, Sotos syndrome, Weaver syndrome	PLAUR (19q13)	Receptor for urokinase plasminogen activator
GRIK2 (6q16.3-q21)	Glutamate receptor, ionotropic, kainate 2, GluR6	DMPK (19q13.3)	Ser/Thr kinase, interact with Rho, Myotonic dystrophy type 1,
AHI1 (6q23.3)	Cerebellar & cortical development, Joubert syndrome	DYRK1A (21q22.13)	Dual-specificity protein kinase, Down syndrome critical region
AUTS2 (7q11.22)	Autism susceptibility candidate 2	TBX1 (22q11.21)	T-box transcription factor, embryonic tissue development
RELN (7q22)	Extracellular matrix protein, neuronal migration & position	ADSL (22q13.1)	Adenylosuccinate lyase, purine synthesis, succinylpurinemic autism
SERPINE1 (7q22.1)	Serpin peptidase inhibitor, class E, member 1; serine protease inhibitor	SHANK3 (22q13.3)	Postsynaptic scaffold protein, synapse function & formation
FOXP2 (7q31)	Forkhead box P2, transcription factor	PTCHD1 (Xp22.11)	Receptor for Shh, neural tube formation & brain development
MET (7q31)	Proto-oncogene, HGF receptor protein Tyr kinase,	NLGN4X (Xp22.32-p22.31)	Cell adhesion, synapse assembly, interact with NRXN1
CADPS2 (7q31.3)	Regulate release of neuropeptides & monamines	CDKL5 (Xp22)	Ser/Thr kinase, X-linked infantile spasm syndrome/Rett syndrome
EN2 (7q36)	Homeobox transcription factor, pattern formation of the CNS	ARX (Xp21)	Transcriptional regulation, CNS development, X-linked mental retardation, epilepsy
CNTNAP2 (7q36.1)	Cell adhesion, interact with K^+^ channel in myelinated axons	IL1RAPL1 (Xp22.1-p21.3)	Interleukin 1 accessary protein like, memory & leaning, X-linked mental retardation
CHD7 (8q12.2)	CHARGE syndrome, DNA helicase, chromatin remodeling	DMD (Xp21.2)	Dystrophin, Duchenne & Becker muscular dystrophy, cytoskeletal protein
FABP5 (8q21.13)	Fatty acid uptake, transport & metabolism	FGD1 (Xp11.21)	Cdc42 GEF, faciogenital dysplasia, X-linked mental retardation
VPS13B (8q22.2)	Cohen syndrome, vesicle transport & protein sorting	NLGN3 (Xq13.1)	Cell adhesion, synapse assembly, interact with NRXN1
TSC1 (9q34)	Tuberous sclerosis protein, regulate Rheb GTPase & mTOR	ATRX (Xq21.1)	Chromatin remodeling, alpha-thelassemia/mental retardation syndrome X-linked,
PTEN (10q23.3)	PIP3dephospholyation, negative regulator of PI3K pathway	FMR1 (Xq27.3)	mRNA trafficking, fragile X mental retardation
DHCR7 (11q13.2-q13.5)	Biosynthesis of cholesterol	AFF2 (Xq28)	Putative transcriptional activator, fragile X E syndrome
SHANK2 (11q13.3-q13.4)	Postsynaptic scaffold protein, synapse function & formation	SLC6A8 (Xq28)	Creatine transporter, X-linked creatine deficiency syndrome
GRIN2B (12p12)	Glutamate receptor, ionotropic, N-methyl D-aspartate 2B	MECP2 (Xq28)	Methyl CpG binding protein 2, transcriptional repression, Rett syndrome, development
CACNA1C (12p13.3)	Voltage-dependent Ca^2+^ channel α 1C subunit (L type)	RPL10 (Xq28)	Ribosomal protein L10, a component of 60S subunit, translation
AVPR1A (12q14-q15)	Vasopressin receptor (Gq/11-PLC-coupled)		

Modified from the data reported in references [5,12,19].

**Figure 1:**
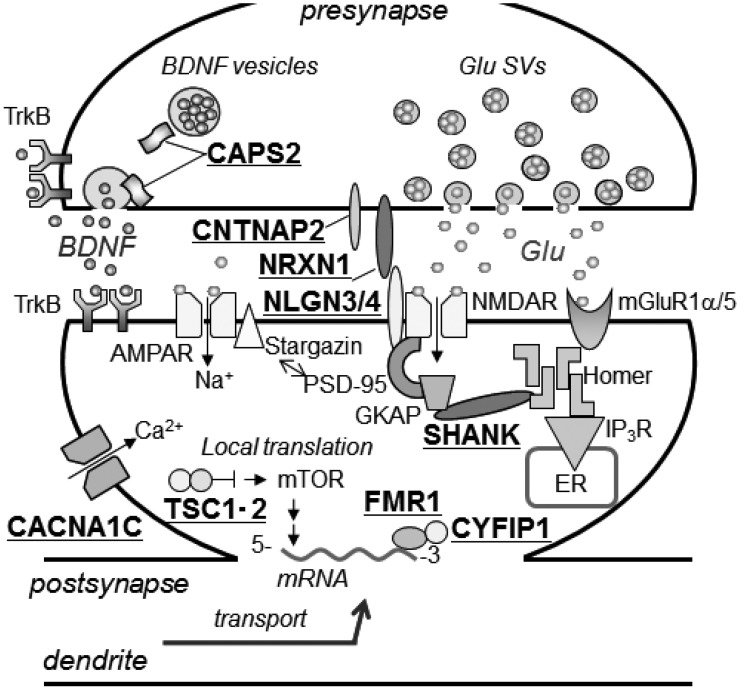
Many ASD-associated genes and gene candidates are involved in synapse function and structure. ASD-associated genes and gene candidates (indicated by underlining) are involved in synaptic connections, synaptic transmission, synaptic plasticity, activity-dependent gene expression, and local translation in synapses.

Many ASD-associated gene candidates code for proteins involved in the development and function of neural circuits (Figure 1). These genes include those involved in neuronal differentiation, migration and circuit formation (*DISC1*, *CDH10*, *CDH9*, *SEMA5A*, *RELN*, *MET*, *PTEN*, and *ITGB3)*, as well as those involved in regulation of synaptic adhesion (*NRXN1*, *CNTNAP2*, *NLGN3*, and *NLGN4X)*, synaptic transmission (*OXTR*, *GRIK2*, *CADPS2*, *CACNA1C*, *AVPR1A*, *SLC6A4*, *GABRB3*, *KCNJ6, and SHANK3)*, and transcription and translation (*SEPRINE*, *EN2*, *TSC1*, *TSC2*, *A2BP1*, *FMR1*, and *MECP2)* [5,12,19]. Recent exome sequencing studies have identified six recurrently-mutated genes (*TBR1*, *TBLXR1*, *CHD8*, *DYRK1A*, *PTEN*, and *GRIN2B*), which were reported to contribute to 1% of sporadic ASD [12]. Because the clinical symptoms vary among ASD patients, it is likely that ASD is not caused by a single gene. Instead, ASD is probably a disorder that involves multiple genes, with an intricate interaction among these genes. It remains unclear what combination of gene mutations and/or variations affect normal brain development. Computational [5] and transcriptomic co-expression [13] analyses of ASD genetics have been performed to identify signaling pathways or networks that incorporate these identified genes, and which will shed light on understanding the mechanisms underlying ASD pathogenesis. For example, GeneMANIA prediction shows that ASD-associated genes form an interaction network based on co-localization, pathways, physical interactions, and predicted relationships (Figure S1, see Online Supplementary information).

### 2.2. Non-Heredity Factors

#### 2.2.1. Environmental Factors

It has been suggested that the risk of developing ASD increases with exposure to certain environmental factors. Ingestion of teratogenic substances (e.g., thalidomide, valproate, and misoprostol) during pregnancy, infection with viruses (e.g., influenza, rubella, and cytomegalovirus) during pregnancy, smoking during the early stage of pregnancy, and advanced age of parents, all increase the risk among children of developing ASD (for reviews, see references [1] and [7]). The risk of developing ASD may increase depending on interactions between genes and environmental factors. Further studies on environmental factors are required.

#### 2.2.2. Epigenetic Factors

Chromatin remodeling (biochemical modifications such as DNA methylation and histone acetylation) is induced during the prenatal and postnatal periods, giving rise to individual differences in transcriptional regulation of genes. It is known that changes in chromatin structure can be passed down through generations (for a review, see reference [6]). This epigenetic information becomes a factor that alters the expression pattern of ASD-associated genes and thereby provides information that differs from inherent genetic information, potentially resulting in a risk for developing ASD [21]. For example, imprinting, in which expression of only one allele derived from one parent (single allele expression) occurs because of inactivation of the other allele by DNA methylation, occurs at ASD-associated chromosomal loci, including 15q11-q13 and 7q21.2-q31.31 [6]. Duplication of the 15q11-q13 locus has frequently been reported in ASD [6]. Therefore, it is thought that interaction between epigenetic heredity factors (e.g., DNA methylation), changes in gene copy number in epigenetic parent-specific regions, and other heredity factors are involved in ASD onset.

## 3. ASD-Related Animal Models

Behavioral phenotypes, including social interaction [22,23,24], the relationship between infant ultrasonic vocalization (USV) and maternal retrieval behavior [25], and restricted repetitive behavior [26], have been evaluated in genetically modified ASD mouse models [2,27]. The influence of ASD-associated genetic variations on synaptic transmission and plasticity has also been receiving attention [28]. These models are typically constructed by introducing an ASD-associated genetic variation or mutation into a mouse homolog, resulting in a mouse that harbors a specific susceptibility gene. ASD animal models without genetic modifications are also possible. These include chemical-induced models of rats and mice, exposed to chemical substances that are prenatal risk factors, or lesion-based models of monkeys and rats, with structural damage induced in specific brain regions such as the cerebellum [2]. Genetically modified animals with single or a combination of multiple gene mutations, are useful for understanding a particular aspect of a hereditary factor. However, as ASD is a multifactorial disorder, it is difficult to construct an animal model that includes every characteristic. 

Moreover, there are differences between rodents and humans with regard to social interaction and communication. Nevertheless, a genetically modified mouse model is important for understanding hereditary factors, despite the limitations in social nature and experimental methods. Mutational influences in mouse models are analyzed with these considerations taken into account.

The following describes *Caps2/Cadps2* gene mutation mice, developed by ourselves and others, as well as a brief introduction to mice with genetic modifications in relatively well-characterized ASD-associated genes.

### 3.1. Caps2/Cadps2 Variant Mice

**Figure 2:**
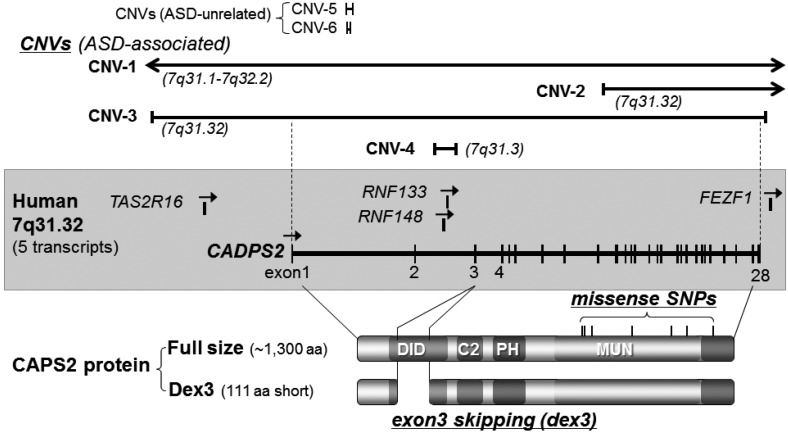
Protein and genomic structure, single-nucleotide polymorphisms, and copy number variations of human *CAPS2/CADPS2*. ***Bottom***, CAPS2 protein structure. There are several alternative splicing variants in mice (see Table 3, [43]). In mouse brain, the major form of CAPS2 protein (full) consists of approximately 1300 amino acid residues (1275–1355 aa). Increased expression of a rare alternative splicing variant lacking exon 3 (deletion of exon 3, dex3) was found in some patients with autism [43]. Exon 3 encodes 111 amino acids. Seven non-synonymous single-nucleotide polymorphisms (SNPs) have been identified within the human *CAPS2/CADPS2* gene. ***Middle***, exon-intron structure of the human *CAPS2/CADPS2* gene located on 7q31.32. Five transcripts (*CAPS2/CADPS2*, *TAS2R16*, *RNF148*, *RNF133*, and *FEZF1*) have been annotated in this region, although *CAPS2/CADPS2* is the only gene with an exon-intron structure and characterized to be expressed as a functional protein. ***Top***, examples of copy number variations (CNVs) affecting *CAPS2/CADPS2* on 7q31.32 (for details, see Table 2). Regions affected by six CNVs of the deletion type are indicated by solid lines (CNV1-6). CNV 1–4 have been identified in ASD patients, but CNV-5 and -6 have no evidence for a connection with ASD. CNV-3 causes deletion of 0.75 Mb, including four genes (*CAPS2/CADPS2*, *TAS2R16*, *RNF148*, and *RNF133*) [16]. CNV-2 causes deletion of 0.94 Mb from exon 9 onwards in *CAPS2/CADPS2* [39]. CNV-1 deletes 15 Mb, affecting about 50 genes including *CAPS2/CADPS2*. CNV-4 causes deletion of 0.044 Mb containing intron 2 of *CAPS2/CADPS2*, and *RNF148* and *RNF133* [18]. CNV-5 [47] and CNV-6 [48] delete short sequences of intron 1 in *CAPS2/CADPS2*. Duplication (0.43 Mb) of *CAPS2/CADPS2* was also reported in ASD [44] (not shown in this figure).

The CAPS (or CADPS) family is a secretory-related protein family that regulates secretory granule exocytosis [29], including monoamines and neuropeptides, as well as Golgi trafficking [30,31,32]. In vertebrates it consists of two genes, *CAPS1/CADPS1* and *CAPS2/CADPS2* (Figure 2). We and others found that CAPS2 kinetically promotes secretion of brain-derived neurotrophic factor (BDNF) [33,34,35]. BDNF is involved in neuronal maturation and synaptic plasticity. Furthermore, it has been suggested that BDNF is involved in psychiatric disorders, such as schizophrenia and depression, and developmental disorders. The human *CAPS2* gene is located within 7q31.32, mapping to the autism susceptibility locus 1 (*AUTS1*:7q31-q33) [36]. In ASD patients, at least 12 CNVs caused by deletion or insertion of fragments containing the *CAPS2* gene locus [1,16,18,37,38,39,40,41,42] (Table 2), and at least 12 single nucleotide variations, which trigger missense, silence, and insertion mutations in the *CAPS2* gene [36,43] (Table 3), have thus far been reported. Some of the CNVs have been identified in multiplex ASD families and one CNV (see Table 2 and CNV-3 in Figure 2) causes deletion of 0.75 Mb, including four genes (*CAPS2*, *TAS2R16*, *RNF148*, and *RNF133*), of which only the *CAPS2* gene consists of a known protein coding sequence with an exon-intron structure [16]. A recent study on exon-disrupting CNVs in 253 autism candidate genes also showed paternally inherited duplication (0.43 Mb) within the *CAPS2* gene in ASD [44] (Table 2). These data suggest the possible association of *CAPS2* variations with ASD. Moreover, by comparing *CAPS2* gene expression patterns between autism patients and healthy controls, it has been shown that the *CAPS2-dex3* type, with deletion of exon 3 (111 amino acids) by rare alternative splicing, is abnormally increased in some autism patients [43]. Interestingly, CAPS2-dex3 expressed in cultured neurons does not accumulate in axons but instead localizes to somatodendritic compartments. Therefore, before the generation of CAPS2-dex3 model mice, it was assumed that localized BDNF secretion from axons and synaptic terminals was not promoted by *CAPS2-dex3*, and thereby affected correct development of synapses and neural circuits.

***Caps2* KO mice:** *Caps2* KO mice are generally born normal and show no significant abnormalities in macroscopic anatomical morphology, basic sensory functions, or locomotor abilities. In KO mice, BDNF secretory activity decreases, and BDNF amounts in the cerebellum, hippocampus, and cerebrum also decrease [43,45]. KO mice show a deficiency in diurnal rhythms of BDNF levels in the neocortex at 14 days of age [46]. A delay in development (proliferation and migration) of granule cells, aplasia of Purkinje cell synapses and dendrites, as well as decreased paired-pulse facilitation at parallel fiber-Purkinje cell synapses, are also observed in the cerebellum [47]. The number of parvalbumin-positive interneurons in the cerebral cortex and hippocampus decreases, although these numbers recover with intraventricular administration of exogenous BDNF [43]. The late phase of long-term potentiation (LTP) decreases at CA3-CA1 synapses in the hippocampus [35]. Furthermore, decreases in GABA inhibitory synapses and miniature inhibitory postsynaptic currents (mIPSCs), as well as abnormalities in hippocampal theta waves are observed. Thus, a disorder of GABAergic inhibitory circuit formation has been postulated. The behavioral traits of KO mice include decreased social interaction and exploratory behavior, increased anxiety-like behaviors in unfamiliar environments, defective intrinsic circadian rhythms, and maternal and nurturing behavior [43]. 

**Table 2:** Copy number variations around the 7q31.3 region.

Chromosome loci	Diagnosis ^(1)^	Gender^(2)^/family ^(3)^	CNV type ^(4)^	CNV size/no. of affected genes	References
7q31.1-q31.31	ASD	M/SPX	loss	25 genes	[40]
7q31.1-q31.32	autism, DVD	M/SPX	loss	11 Mb/>50 genes	[37]
7q31.1-q32.2	ASD, DVD	F/SPX	loss	15.49 Mb/77 genes	[40]
7q31.2-q32	ASD, DVD	M/SPX	loss	26 Mb/	[37]
7q31.2-q32.2	autism, DVD, Williams syndrome	F/SPX	loss	15 Mb/>50 genes	[37,38]
7q31.3	ASD	-/MPX	loss	1.52 Mb/2 genes	[18]
7q31.3	ASD	M/SPX	loss	0.044 Mb/intron 2 of *CADPS2*	[18]
7q31.31-q31.33	ASD	M/-	loss	5.4 Mb	[41]
7q31.32	ASD	-/MPX	loss	0.75 Mb/4 genes	[9]
7q31.32	ASD	-/MPX	loss	0.94 Mb/4 genes	[39]
7q31.32	ASD	-/-	loss	1.52 Mb/7 genes	[42]
7q31.32-q34	autism	M/-	gain		[1]
7q31.3	ASD	-/-	gain	0.43 Mb/exon of *CADPS2*	[44]

Basic information was obtained from AutDB [49] and the Autism Chromosome Rearrangement Database [50]. ^(1)^ ASD, autism spectrum disorder; DVD, developmental verbal dyspraxia. ^(2)^ Gender: M, male; F, female; -, unknown. ^(3)^ Family: MPX, multiplex; SPX, simplex; -, unknown. ^(4)^ CNV: loss, deletion; gain, duplication.

**Table 3:** Rare sequence variants (SNPs) of *CADPS2* at 7q31.3.

Variant Type	Allele Change	Residue Change	Reference
Missense	T2405C	I761T	[43]
Missense	G2419C	V766L	[43]
Missense	C2504A	T794N	[43]
Missense	A2896G	T925A	[43]
Missense	G3286A	D1055N	[43]
Missense	G3286A	D1055N	[43]
Missense	G3457A	D1112N	[43]
Missense	C3722T	T1200M	[43]
Missense	G983A	Ala297Thr	[43]
Silent	C2461T	N/A **	[36]
Silent	A2539C	N/A	[36]
Insertion	N/A	N/A	[36]

AutDB (Autism Database [51]) was used to obtain the above information; ** N/A, not available.

***Caps2* heterozygous mice:** *Caps2* heterozygous mice model a deletion-type CNV of *CAPS2* in ASD patients (Table 2). In performance levels of maternal nurturing behavior and unfamiliar environment-based anxiety tests, heterozygotes show almost intermediate levels between wild-type and KO (homozygote) mice [43,52]. Although heterozygotes show a decreased number of USV calls at P5 after maternal separation, and longer intrinsic circadian rhythms under constant dark conditions, compared with wild-types, they are not significantly different from wild types in social interaction with an unfamiliar mouse at adulthood [52]. These results suggest that ASD-associated anxiety disorder can develop in people with ASD, a disorder involving multiple factors, because of *CAPS2* gene deleted-type CNV, whereas significant social behavior impairment does not. Therefore, it is predicted that the risk of developing symptoms defining ASD increases through a combination effect of *CAPS2* CNVs and other factors.

***Caps2-dex3* mice**: In mice, the gene homologous to human *CAPS2* is located on chromosome 6. Among the 28 exons of the *Caps2* gene, a *Caps2-dex3* mouse with specific deletion of exon 3 was constructed, generating a mouse model expressing a rare splicing subtype identical to human *CAPS2-dex3* [53], and which was increased in some of human ASD patients [53] (Figure 2). This mouse mutant is born approximately within the normal birth rate and shows no significant visible differences compared with wild-type mice. A shortened *CAPS2-dex3*, lacking the 111 amino acid residues encoded by exon 3, was found rarely in regions with axons concentrated in the cerebral cortex, hippocampus, and cerebellar cortex, where *CAPS2-dex3* is mostly detected in the cell body or dendrite. As predicted, BDNF secretion by the axon and presynapse was decreased. Furthermore, an effect on excitatory synapse morphology and differentiation of some inhibitory neurons was apparent, with decreased social interaction, social novelty recognition, and the capability to adapt to new environments, and increased anxiety, impairment of maternal nurturing behavior, and circadian rhythm abnormalities also observed. These results show that CAPS2 is necessary to promote subcellular compartment (axon *vs*. somato-dendrite)-specific secretion of BDNF. Increased CAPS2-dex3 expression reduces BDNF secretion in axons, and disturbs local BDNF secretion patterns. Therefore, we suggest that increased CAPS2-dex3 expression leads to increased risk for developing ASD-like behaviors [43,53].

As indicated by *CAPS2* gene mutant mice, disturbances in the CAPS2-regulated secretory granule pathway, affects enhanced release and/or polarized subcellular release of neuropeptide(s) such as BDNF, and possibly monoamine(s), and causes impairments in the development and function of synapses and/or circuits (particularly inhibitory pathways), resulting in ASD-like behavioral traits. With regard to the role of the CAPS family in regulating monoamine and neuropeptide release, it is notable that some monoamine- and neuropeptide-related genes are also ASD gene candidates (Table 1): *SLC6A4* is a plasma membrane 5-HT receptor, *AVPR1A* and *OXTR* are receptors for the neuropeptides vasopressin and oxytocin, respectively. Moreover, *CAPS2* KO mice impairments in the GABAergic system and BDNF secretion may be related to the other ASD gene candidates (Table 1), such as the *GABRB3* gene, encoding the GABAA receptor β-3 subunit, and the *MeCP2* gene, the causative gene of Rett syndrome and encoding a methyl-CpG-binding protein implicated in transcriptional regulation of the *BDNF* gene.

### 3.2. Other ASD-Associated Gene Modified Mice

In this section, we focus on well-characterized or representative mouse models, with regard to synaptic connectivity (neurexins and neuroligins), formation of the postsynaptic protein signaling complex (shank), and neuropeptide regulation related to social behavior (oxytocin and related molecules). We have also included a model of the 15q11-13 duplication, a chromosomal mutation that frequently occurs in ASD. For other genes, such as *PTEN* and *CNTNAP2*, see References [1,2,4,5,22,23,24,25,26,27].

#### 3.2.1. Mediating Adhesion between Pre- and Post-Synaptic Membranes

Neuroligin (Nlgn) and neurexin (Nrxn) are adhesion transmembrane proteins that localize to the post- and pre-synaptic membranes, respectively, and are involved in connecting the pre- and post-synapse. The cytoplasmic domain of Nlgn binds to PSD-95, a scaffold protein for postsynaptic density (PSD) proteins such as the NMDA-type glutamate receptor. In humans, the *NLGN* gene family comprises five genes (*NLGN1-4*, *4Y*). A non-synonymous SNP (R451C) [54] in *NLGN3* (Xq13.1), and a non-synonymous SNP (R704C) [55] and frame-shift mutation [54] (causing the amino acid sequence to terminate prematurely at amino acid residue 396) (396X) in *NLGN4* (Xp22.32-p22.31) have been reported. In humans, the *NRXN* gene family comprises three genes (*NRXN1-3*), encoding long α- and short β-neurexins that are synthesized depending on the promoter. A CNV that affects the *NRXN1α* gene (2p16.3) has been associated with ASD.

***Nlgn3 (R451C)* mice**: Decreased social interaction and increased performance in the Morris water maze task were observed in *Nlgn3* (R451C) mice [56]. Cell morphology changes and behavioral phenotypes are rarely observed in *Nlgn3* KO mice. The observed phenotypes are suggested to be due to a gain-of function [56]. However, studies using a different *Nlgn3* (R451C) mouse line, constructed independently, reported no abnormalities in social interaction [57]. Therefore, assessment of human-like behavior using this mouse model may not be straightforward [22]. 

***Nlgn4* KO mice**: No marked changes in basic sense, recognition, locomotion, learning, or memory were found. However, decreased social interaction, USV produced by male KO mice cohabiting with sexually excited female mice, and brain mass were observed [58].

***Nrxn1α* KO mice**: In *Nrxn1α* KO mice, miniature excitatory postsynaptic currents (mEPSCs) and excitatory postsynaptic currents were decreased [59]. No obvious changes in social interaction and anxiety were observed, although the amount of grooming behavior increased and nesting capability decreased. Intraperitoneal administration of *D*-cycloserine, a partial agonist of NMDAR, restores grooming behavior [60]. 

These results suggest that impairments in synaptic adhesion, due to interactions between NLGN and NRXN, induce abnormalities in functioning of synapses and circuits, resulting in ASD-like phenotypes dependent on the type of synaptic adhesion molecules affected. 

#### 3.2.2. Tethering of Signal Transduction-Associated Molecules in the Postsynaptic Density

Shank protein, a scaffold protein located in the PSD of excitatory synapses, binds PSD proteins, including NLGN, GKAP, and Homer, and plays a role in tethering NMDA-type glutamate receptor (NMDAR) complexes (NMDAR—PSD-95—GKAP) and type 1 metabotropic glutamate receptor (mGluR) complexes (mGluR1α/5—Homer) to the PSD. The human *SHANK* gene family comprises three genes *(SHANK1-3)*. A CNV, frame-shift mutation, and non-synonymous SNP within the *SHANK3* (22q13.3) gene have been discovered in ASD and schizophrenic patients [61]. Similarly, a CNV causing deletion of either exons 6 and 7 or exon 7 alone, and a non-synonymous SNP, of *SHANK2* (11q13.3-q13.4) have been reported in ASD [62]. A CNV causing deletion of the *SHANK1* (19q13.3) gene has also been found [63].

***Shank3* KO mice:** *Shank3* KO mice show pronounced skin lesions due to excessive/repetitive grooming (a type of stereotyped behavior), decreased social interaction, and abnormal social novelty recognition [64]. Interestingly, this model exhibits hypertrophy, and reduced spine density and PSD length, in striatal medium spiny neurons, leading to reduced synaptic transmission within corticostriatal circuitry. 

***Shank2* KO mice**: *Shank*2 KO mice, show decreased social interaction behavior (including USV), and increased repeated jumping (a type of stereotyped behavior) [65]. Decreased NMDAR function is also observed, although it normalizes after *D*-cycloserine administration, resulting in improved social interaction. Furthermore, NMDAR function normalization and social interaction are observed after administration of CDPPB, a positive allosteric mGluR5 modulator [65].

***Shank1* KO mice***: Shank1* KO mice show significantly decreased locomotor capabilities, enhanced anxiety-like behavior, and decreased fear conditioning behavior and maintenance of spatial learning [66].

These observations suggest deficiencies of SHANK-mediated tethering of PSD proteins are associated with ASD risk, and differences in cell types expressing specific SHANK family proteins reflect symptoms. 

#### 3.2.3. Human Chromosome 15q11-13 Repeats

The chromosomal 15q-q13 region repeats at a relatively high frequency in ASD, and contains the ASD-associated genes, *UBE3A* and *GABRB3*. This region also contains a gene cluster that can be DNA methylated, with only a single allele (either maternal or paternal) transcribed. Otherwise, both alleles are transcribed [6]. 

**15q11-13 duplication mice**: In mice containing a duplication of the 6.3-Mb region of 7C on chromosome 7 (corresponding to human 15q11-q13), the transcription level of the region changes depending on if the duplication is paternally (*patDp/+*) or maternally (*matDp/+*) derived, and if the duplication is methylated [67]. For example, expression of the *Ube3a* gene, transcribed from the maternal allele, increases 2-fold in *matDp/+* mice compared with wild-type mice. In contrast, expression of the *Gabrb3* gene, transcribed from both alleles, is highly increased (with an amount corresponding to three copies) when not methylated in *patDp/+* and *matDp/+* mice. Decreases in social interaction and behavioral flexibility (preservative tendency), as well as increased USV abnormalities in neonatal mice during the preweaning period, and increased anxiety, are observed in *patDp/+* mice. However, no social interaction abnormalities are observed in *matDp/+* mice. Moreover, snoRNA MBII52 expression, transcribed from a paternal allele, increases in the brains of *patDp/+* mice. Because of the MBII52 deletion in Prader-Willi syndrome, it is known that MBII52 affects RNA editing, and decreases alternative splicing (and serotonin activity), of the serotonin 5-HT_2c_ receptor. Ca^2+^ reactivity elicited by a 5-HT_2c_ receptor agonist is increased in cultured neurons derived from *patDp/+* mice, and serotonin levels in the brain during and after developmental stages are low in *patDp/+* mice. Exploratory behavior in unfamiliar environments is also decreased [68]. 

This mouse model is useful for understanding the symptoms of patients with 15q11-q13 duplications, and studying epigenetic relationships.

#### 3.2.4. Secretory Pathways of Neuropeptide Hormones and Oxytocin

CD38 is a membrane protein involved in biosynthesis of cyclic ADP ribose (cADPR), which acts as an intracellular messenger to induce Ca^2+^ release from intracellular Ca^2+^ stores [69]. cADPR-induced Ca^2+^ increases trigger secretion of oxytocin (OXT), a peptide hormone. OXT is associated with amicable behaviors, including reproduction, pair formation, maternal/paternal behavior, and social interaction [70,71]. A *CD38* gene (4p15) containing a SNP, a relatively large sequence of a CNV carrying the *OXT* gene (20p13), and an OXT receptor (*OXTR*) gene (3p25) containing a SNP and CNV have been found in ASD patients [69].

***Cd38* KO mice**: Defects in nurturing behavior by female mice and in social interaction by male mice are observed in *Cd38* KO mice [72]. Furthermore, maternal segregation results in hyperactivity and low USV number in KO pups. Blood serum OXT levels are decreased, and behavioral abnormalities can be restored by external OXT administration.

***Oxt* and *Oxtr* KO mice**: It has been reported that both *Oxt* and *Oxtr* KO mice, exhibit behavioral disorders similar to those observed in *Cd38* KO mice [22,69]. 

Based on studies using these model mice, it has been suggested that the signal transduction pathway of CD38-OXT-OXTR is particularly involved in communication between individuals. A missense SNP (R140W) in *CD38* was identified in a small Japanese population and shown to be associated with ASD [73]. Infection with lentiviral vectors containing wild-type CD38, but not CD38-R140W, rescues the social recognition deficit of CD38 KO mice, suggesting R140W may be associated with social behavior [72]. Interestingly, ASD patients with the R140W SNP have low plasma OXT levels, and the symptoms of a proband could be ameliorated by treatment with nasal OXT spray [73]. Results from clinical trials using intranasal OXT administration are anticipated to provide possible hope for ASD treatment [71]. 

#### 3.2.5. Mouse Models for Disorders Involving a Single Gene Accompanied by Autism Symptoms

Fragile-X syndrome, Rett syndrome, and tuberous sclerosis are thought to be single gene disorders with mutations in the *FMR1*, *MECP2*, and *TSC1/TSC2* genes, respectively. Patients affected by these disorders also show autism-like symptoms. Thus, mice with mutations in these genes are useful models for ASD (for reviews, see [2,22]).

## 4. Conclusions

Multiple factors originating from genetics, epigenetics, and the environment are associated with the onset and risk of ASD, and thought to be related to variations in ASD symptoms. Animal models with ASD-associated gene mutations or variations are useful for analyzing the role and involvement of heredity factors in ASD. It is anticipated that further research and development using mutant mice constructed with many other ASD-associated gene candidates and their mutations and variations, as well as further research on environmental factors that impact ASD, will enable a better understanding of the molecular mechanisms involved in ASD pathogenesis. Moreover, it will be necessary to develop models using animals with greater social skills that better reflect the characteristics of ASD. 

Discovery and development of reagents that control any step in the CAPS2-mediated BDNF pathway may be valuable for further development of tools for CAPS2-associated ASD diagnoses or cure. Furthermore, studies of various mouse models with mutations in ASD gene candidates will contribute to specific diagnoses and treatments for ASD. Collectively, combining the results of ASD mouse models and clinical outcomes will open new windows towards a cure for ASD.

## Supplementary Files

Supplementary File 1 Supplementary Information (PDF, 417 KB)

## References

[1] Folstein S.E., Rosen-Sheidley B. (2001). Genetics of autism: Complex aetiology for a heterogeneous disorder. Nat. Rev. Genet..

[2] DiCicco-Bloom E., Lord C., Zwaigenbaum L., Courchesne E., Dager S.R., Schmitz C., Schultz R.T., Crawley J., Young L.J. (2006). The developmental neurobiology of autism spectrum disorder. J. Neurosci..

[3] American Psychiatric Association (2013). Diagnostic and Statistical Manual of Mental Disorders.

[4] Abrahams B.S., Geschwind D.H. (2008). Advances in autism genetics: On the threshold of a new neurobiology. Nat. Rev. Genet..

[5] Bill B.R., Geschwind D.H. (2009). Genetic advances in autism: Heterogeneity and convergence on shared pathways. Curr. Opin. Genet. Dev..

[6] Schanen N.C. (2006). Epigenetics of autism spectrum disorders. Hum. Mol. Genet..

[7] Grabrucker A.M. (2013). Environmental factors in autism. Front. Psych..

[8] Neale B.M., Kou Y., Liu L., Ma'ayan A., Samocha K.E., Sabo A., Lin C.F., Stevens C., Wang L.S., Makarov V. (2012). Patterns and rates of exonic de novo mutations in autism spectrum disorders. Nature.

[9] O’Roak B.J., Deriziotis P., Lee C., Vives L., Schwartz J.J., Girirajan S., Karakoc E., Mackenzie A.P., Ng S.B., Baker C. (2011). Exome sequencing in sporadic autism spectrum disorders identifies severe de novo mutations. Nat. Genet..

[10] O’Roak B.J., Vives L., Girirajan S., Karakoc E., Krumm N., Coe B.P., Levy R., Ko A., Lee C., Smith J.D. (2012). Sporadic autism exomes reveal a highly interconnected protein network of de novo mutations. Nature.

[11] Sanders S.J., Murtha M.T., Gupta A.R., Murdoch J.D., Raubeson M.J., Willsey A.J., Ercan-Sencicek A.G., DiLullo N.M., Parikshak N.N., Stein J.L. (2012). *De novo* mutations revealed by whole-exome sequencing are strongly associated with autism. Nature.

[12] O’Roak B.J., Vives L., Fu W., Egertson J.D., Stanaway I.B., Phelps I.G., Carvill G., Kumar A., Lee C., Ankenman K. (2012). Multiplex targeted sequencing identifies recurrently mutated genes in autism spectrum disorders. Science.

[13] Voineagu I., Wang X., Johnston P., Lowe J.K., Tian Y., Horvath S., Mill J., Cantor R.M., Blencowe B.J., Geschwind D.H. (2011). Transcriptomic analysis of autistic brain reveals convergent molecular pathology. Nature.

[14] Weiss L.A., Arking D.E., Daly M.J., Chakravarti A.A. (2009). Genome-wide linkage and association scan reveals novel loci for autism. Nature.

[15] Sebat J., Lakshmi B., Malhotra D., Troge J., Lese-Martin C., Walsh T., Yamrom B., Yoon S., Krasnitz A., Kendall J. (2007). Strong association of de novo copy number mutations with autism. Science.

[16] Szatmari P., Paterson A.D., Zwaigenbaum L., Roberts W., Brian J., Liu X.Q., Vincent J.B., Skaug J.L., Thompson A.P., The Autism Genome Project Consortium (2007). Mapping autism risk loci using genetic linkage and chromosomal rearrangements. Nat. Genet..

[17] Cook E.H., Scherer S.W. (2008). Copy-number variations associated with neuropsychiatric conditions. Nature.

[18] Bucan M., Abrahams B.S., Wang K., Glessner J.T., Herman E.I., Sonnenblick L.I.,  Retuerto A.A., Imielinski M., Hadley D., Bradfield J.P. (2009). Genome-wide analyses of exonic copy number variants in a family-based study point to novel autism susceptibility genes. PLoS Genet..

[19] Pinto D., Pagnamenta A.T., Klei L., Anney R., Merico D., Regan R., Conroy J., Magalhaes T.R., Correia C., Abrahams B.S. (2010). Functional impact of global rare copy number variation in autism spectrum disorders. Nature.

[20] Kong A., Frigge M.L., Masson G., Besenbacher S., Sulem P., Magnusson G., Gudjonsson S.A., Sigurdsson A., Jonasdottir A., Jonasdottir A. (2012). Rate of de novo mutations and the importance of father’s age to disease risk. Nature.

[21] Persico A.M., Bourgeron T. (2006). Searching for ways out of the autism maze: Genetic, epigenetic and environmental clues. Trends Neurosci..

[22] Silverman J.L., Yang M., Lord C., Crawley J.N. (2010). Behavioural phenotyping assays for mouse models of autism. Nat. Rev. Neurosci..

[23] Moy S.S., Nadler J.J. (2008). Advances in behavioral genetics: Mouse models of autism. Mol. Psych..

[24] Yang M., Weber M.D., Crawley J.N. (2008). Light phase testing of social behaviors: Not a problem. Front. Neurosci..

[25] Hahn M.E., Lavooy M.J. (2005). A review of the methods of studies on infant ultrasound production and maternal retrieval in small rodents. Behav. Genet..

[26] Lewis M.H., Tanimura Y., Lee L.W., Bodfish J.W. (2007). Animal models of restricted repetitive behavior in autism. Behav. Brain Res..

[27] Crawley J.N. (2008). Behavioral phenotyping strategies for mutant mice. Neuron.

[28] Shinoda Y., Sadakata T., Furuichi T. (2013). Animal models of Autism Spectrum Disorder (ASD): A synaptic-level approach to autistic-like behavior in mice. Exp. Anim..

[29] Berwin B., Floor E., Martin T.F. (1998). CAPS (mammalian UNC-31) protein localizes to membranes involved in dense-core vesicle exocytosis. Neuron.

[30] Sadakata T., Shinoda Y., Sekine Y., Saruta C., Itakura M., Takahashi M., Furuichi T. (2010). Interaction of calcium-dependent activator protein for secretion 1 (CAPS1) with the class II ADP-ribosylation factor small GTPases is required for dense-core vesicle trafficking in the trans-Golgi network. J. Biol. Chem..

[31] Sadakata T., Sekine Y., Oka M., Itakura M., Takahashi M., Furuichi T. (2011). Calcium-dependent activator protein for secretion 2 interacts with the class II ARF small GTPases and regulates dense-core vesicle trafficking. FEBS J..

[32] Sadakata T., Kakegawa W., Shinoda Y., Hosono M., Katoh-Semba R., Sekine Y., Sato Y., Tanaka M., Iwasato T., Itohara S., Furuyama K., Kawaguchi Y., Ishizaki Y., Yuzaki M., Furuichi T. (2013). CAPS1 deficiency perturbs dense-core vesicle trafficking and Golgi structure and reduces presynaptic release probability in the mouse brain. J. Neurosci..

[33] Sadakata T., Mizoguchi A., Sato Y., Katoh-Semba R., Fukuda M., Mikoshiba K., Furuichi T. (2004). The secretory granule-associated protein CAPS2 regulates neurotrophin release and cell survival. J. Neurosci..

[34] Sadakata T., Furuichi T. (2008). Developmentally regulated Ca^2+^-dependent activator protein for secretion 2 (CAPS2) is involved in BDNF secretion and is associated with autism susceptibility. Cerebellum.

[35] Shinoda Y., Sadakata T., Nakano K., Katoh-Semba R., Kinameri E., Furuya A., Yanagawa Y., Hirase H., Furuichi T. (2011). Calcium-dependent activator protein for secretion 2 (CAPS2) promotes BDNF secretion and is critical for the development of GABAergic interneuron network. Proc. Natl. Acad. Sci. USA.

[36] Cisternas F.A., Vincent J.B., Scherer S.W., Ray P.N. (2003). Cloning and characterization of human CADPS and CADPS2, new members of the Ca^2+^-dependent activator for secretion protein family. Genomics.

[37] Feuk L., Kalervo A., Lipsanen-Nyman M., Skaug J., Nakabayashi K., Finucane B., Hartung D., Innes M., Kerem B., Nowaczyk M.J. (2006). Absence of a paternally inherited FOXP2 gene in developmental verbal dyspraxia. Amer. J. Hum. Genet..

[38] Zeesman S., Nowaczyk M.J., Teshima I., Roberts W., Cardy J.O., Brian J., Senman L., Feuk L., Osborne L.R., Scherer S.W. (2006). Speech and language impairment and oromotor dysparaxia due to deletion of 7q31 that involves FOXP2. Amer. J. Med. Genet. A.

[39] Christian S.L., Brune C.W., Sudi J., Kumar R.A., Liu S., Karamohamed S., Badner J.A., Matsui S., Conroy J., McQuaid D. (2008). Novel submicroscopic chromosomal abnormalities detected in autism spectrum disorder. Biol. Psychiat..

[40] Marshall C.R., Noor A., Vincent J.B., Lionel A.C., Feuk L., Skaug J., Shago M., Moessner R., Pinto D., Ren Y. (2008). Structural variation of chromosomes in autism spectrum disorder. Amer. J. Hum. Genet..

[41] Okamoto N., Hatsukawa Y., Shimojima K., Yamamoto T. (2011). Submicroscopic deletion in 7q31 encompassing CADPS2 and TSPAN12 in a child with autism spectrum disorder and PHPV. Amer. J. Med. Genet. Part A.

[42] Gai X., Xie H.M., Perin J.C., Takahashi N., Murphy K., Wenocur A.S., D'arcy M., O'Hara R.J., Goldmuntz E., Grice D.E. (2012). Pare structural variation of synapse and neurotransmission genes in autism. Mol. Psychiatry.

[43] Sadakata T., Washida M., Iwayama Y., Shoji S., Sato Y., Ohkura T., Katoh-Semba R., Nakajima M., Sekine Y., Tanaka M. (2007). Autistic-like phenotypes in Cadps2-knockout mice and aberrant CADPS2 splicing in autistic patients. J. Clin. Invest..

[44] Girirajan S., Dennis M.Y., Baker C., Malig M., Coe B.P., Campbell C.D., Mark K., Vu T.H., Alkan C., Cheng Z. (2013). Refinementt and discovery of new hotspots of copy-number variation associated with autism spectrum disorder. Am. J. Hum. Genet..

[45] Sadakata T., Kakegawa W., Mizoguchi A., Washida M., Katoh-Semba R., Shutoh F., Okamoto T., Nakashima H., Kimura K., Tanaka M. (2007). Impaired cerebellar development and function in mice lacking CAPS2, a protein involved in neurotrophin release. J. Neurosci..

[46] Hamatake M., Miyazaki N., Sudo K., Matsuda M., Sadakata T., Furuya A., Ichisaka S., Hata Y., Nakagawa C., Nagata K. (2011). Phase advance of the light-dark cycle perturbs diurnal rhythms of brain-derived neurotrophic factor and neurotrophin-3 protein levels, which reduces synaptophysin-positive presynaptic terminals in the cortex of juvenile rats. J. Biol. Chem..

[47] Bentley D.R., Balasubramanian S., Swerdlow H.P., Smith G.P., Milton J., Brown C.G., Hall K.P., Evers D.J., Barnes C.L., Bignell H.R. (2008). Accurate whole human genome sequencing using reversible terminator chemistry. Nature.

[48] Levy S., Sutton G., Ng P.C., Feuk L., Halperm A.L., Walenz B.P., Axelrod N., Huang J., Kirkness E.F., Deisov G. (2007). The diploid genome sequence of an individual human. PLoS Biol..

[49] AutDB. http://autism.mindspec.org/autdb/CNVHome.do.

[50] Autism Chromosome Rearrangement Database. http://projects.tcag.ca/autism/.

[51] Autism Database. http://autism.mindspec.org/autdb/.

[52] Sadakata T., Shinoda Y., Oka M., Sekine Y., Furuichi T. (2013). Autistic-like behavioral phenotypes in a mouse model with copy number variation of the CAPS2/CADPS2 gene. FEBS Lett..

[53] Sadakata T., Shinoda Y., Oka M., Sekine Y., Sato Y., Saruta C., Miwa H., Tanaka M., Itohara S., Furuichi T. (2012). Reduced axonal localization of a Caps2 splice variant impairs axonal release of BDNF and causes autistic-like behavior in mice. Proc. Natl. Acad. Sci. USA.

[54] Jamain S., Quach H., Betancur C., Rastam M., Colineaux C., Gillberg I.C., Soderstrom H., Giros B., Leboyer M., Gillberg C. (2003). Mutations of the X-linked genes encoding neuroligins NLGN3 and NLGN4 are associated with autism. Nat. Genet..

[55] Yan J., Oliveira G., Coutinho A., Yang C., Feng J., Katz C., Sram J., Bockholt A., Jones I.R., Craddock N. (2005). Analysis of the neuroligin 3 and 4 genes in autism and other neuropsychiatric patients. Mol. Psychiatry.

[56] Tabuchi K., Blundell J., Etherton M.R., Hammerm R.E., Liu X., Powell C.M., Südhof T.C. (2009). A neuroligin-3 mutation implicated in autism increases inhibitory synaptic transmission in mice. Science.

[57] Chadman K.K., Gong S., Scattoni M.L., Boltuck S.E., Gandhy S.U., Heintz N., Crawley J.N. (2008). Minimal aberrant behavioral phenotypes of neuroligin-3 R451C knockin mice. Autism Res..

[58] Jamain S., Radyushkin K., Hammerschmidt K., Ferrante A., Ricceri L. (2008). Reduced social interaction and ultrasonic communication in a mouse model of monogenic heritable autism. Proc. Natl. Acad. Sci. USA.

[59] Etherton M.R., Blaiss C.A., Powell C.M., Südhof T.C. (2009). Mouse neurexin-1alpha deletion causes correlated electrophysiological and behavioral changes consistent with cognitive impairments. Proc. Natl. Acad. Sci. USA.

[60] Blundell J., Blaiss C.A., Etherton M.R., Espinosa F., Tabuchi K., Walz C., Bolliger M.F., Südhof T.C., Powell C.M. (2010). Neuroligin-1 deletion results in impaired spatial memory and increased repetitive behavior. J. Neurosci..

[61] Durand C.M., Betancur C., Boeckers T.M., Bockmann J., Chaste P., Fauchereau F., Nygren G., Rastam M., Gillberg I.C., Anckarsäter H. (2007). Mutations in the gene encoding the synaptic scaffolding protein SHANK3 are associated with autism spectrum disorders. Nat. Genet..

[62] Berkel S., Marshall C.R., Weiss B., Howe J., Roeth R., Moog U., Endris V., Roberts W., Szatmari P., Pinto D. (2010). Mutations in the SHANK2 synaptic scaffolding gene in autism spectrum disorder and mental retardation. Nat. Genet..

[63] Sato D., Lionel A.C., Leblond C.S., Prasad A., Pinto D., Walker S., O'Connor I., Russell C., Drmic I.E., Hamdan F.F. (2012). SHANK1 deletions in males with autism spectrum disorder. Am. J. Hum. Genet..

[64] Peça J., Feliciano C., Ting J.T., Wang W., Wells M.F., Venkatraman T.N., Lascola C.D., Fu Z., Feng G. (2011). Shank3 mutant mice display autistic-like behaviours and striatal dysfunction. Nature.

[65] Won H., Lee H.R., Gee H.Y., Mah W., Kim J.I., Lee J., Ha S., Chung C., Jung E.S., Cho Y.S. (2012). Autistic-like social behaviour in Shank2-mutant mice improved by restoring NMDA receptor function. Nature.

[66] Hung A.Y., Futai K., Sala C., Valtschanoff J.G., Ryu J., Woodworth M.A., Kidd F.L., Sung C.C., Miyakawa T., Bear M.F. (2008). Smaller dendritic spines, weaker synaptic transmission, but enhanced spatial learning in mice lacking Shank1. J. Neurosci..

[67] Nakatani J., Tamada K., Hatanaka F., Ise S., Ohta H., Inoue K., Tomonaga S., Watanabe Y., Chung Y.J., Banerjee R. (2009). Abnormal behavior in a chromosome-engineered mouse model for human 15q11–13 duplication seen in autism. Cell.

[68] Tamada K., Tomonaga S., Hatanaka F., Nakai N., Takao K., Miyakawa T., Nakatani J., Takumi T. (2010). Decreased exploratory activity in a mouse model of 15q duplication syndrome; implications for disturbance of serotonin signaling. PLoS One.

[69] Higashida H., Yokoyama S., Munesue T., Kikuchi M., Minabe Y., Lopatina O. (2011). CD38 gene knockout juvenile mice: A model of oxytocin signal defects in autism. Biol. Pharm. Bull..

[70] Donaldson Z.R., Young L.J. (2008). Oxytocin, vasopressin, and the neurogenetics of sociality. Science.

[71] Meyer-Lindenberg A., Domes G., Kirsch P., Heinrichs M. (2011). Oxytocin and vasopressin in the human brain: Social neuropeptides for translational medicine. Nat. Rev. Neurosci..

[72] Jin D., Liu H.X., Hirai H., Torashima T., Nagai T., Lopatina O., Shnayder N.A., Yamada K., Noda M., Seike T. (2007). CD38 is critical for social behavior by regulating oxytocin secretion. Nature.

[73] Munesue T., Yokoyama S., Nakamura K., Anitha A., Yamada K., Hayashi K., Asaka T., Liu H.X., Jin D., Koizumi K. (2010). Two genetic variants of CD38 in subjects with autism spectrum disorder and control. Neurosci. Res..

