# Supplementary material for: Mouse Models of Mutations and Variations in Autism Spectrum Disorder-Associated Genes: Mice Expressing Caps2/Cadps2 Copy Number and Alternative Splicing Variants
Source: Int J Environ Res Public Health. 2013 Nov 27;10(12):6335–53. doi: 10.3390/ijerph10126335 (PMC3881117; doi:10.3390/ijerph10126335)
Supplement: Supplementary File 1 — Supplementary Information (PDF, 417 KB) [file ijerph-10-06335-s001.pdf]

Mouse Models of Mutations and Variations in Autism Spectrum Disorder-Associated Genes: Mice Expressing *Caps2/Cadps2* Copy Number and Alternative Splicing Variants

Figure S1. A predicted ASD-associated gene network. The network was predicted by the basic network weighting method of GeneMANIA [1] using information on co-localization, pathways, physical interactions and predicted relationships [2]. CADPS2 is highlighted by a red circle. ASD-associated genes are indicated by large black circles. Genes indicated by small gray circles are genes relevant to or functionally interacting with ASD-associated genes.

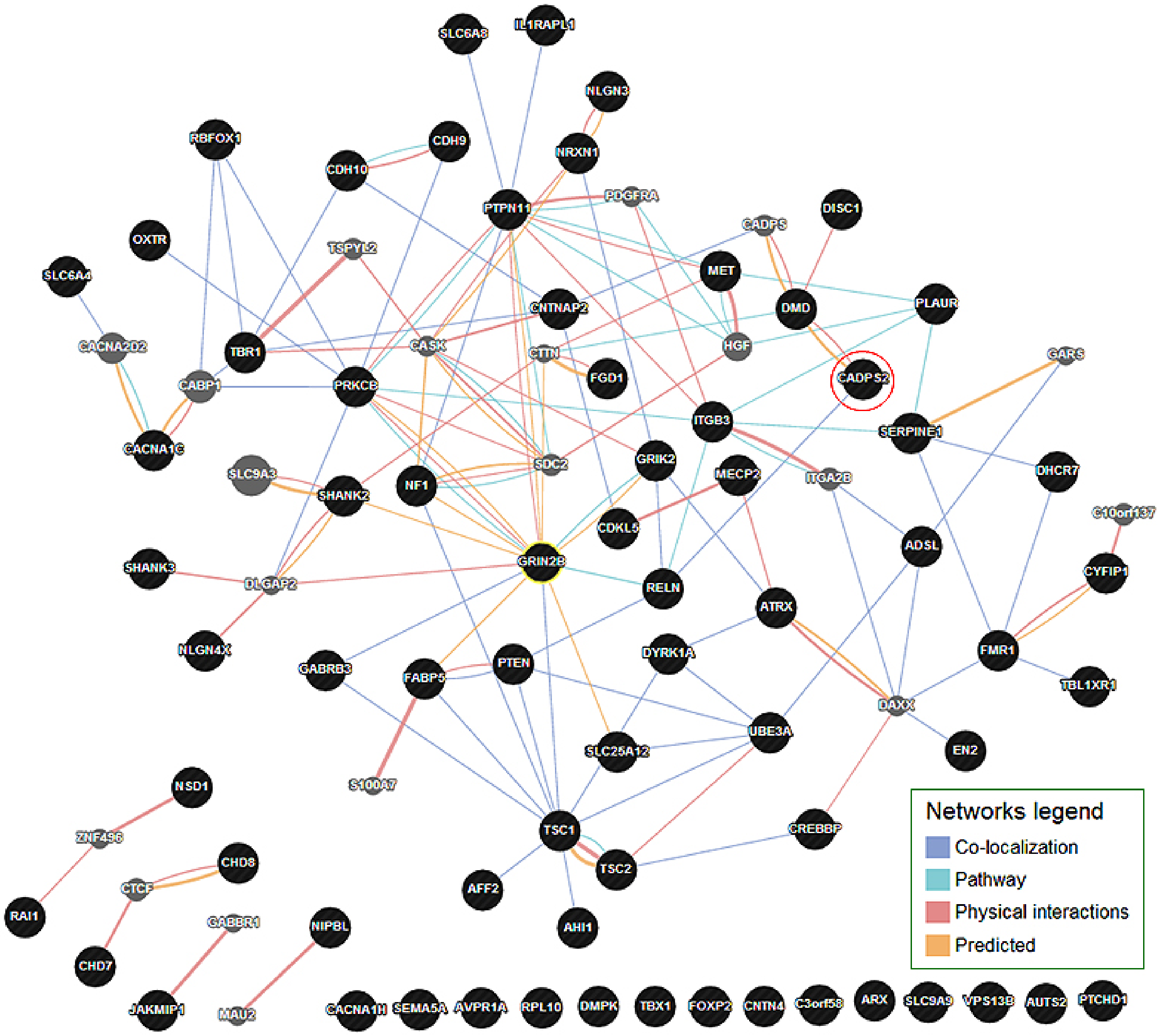

Reference

1. GeneMANIA. Availabel online: http://www.genemania.org (accessed on 15 November 2013).
2. Warde-Farley, D.; Donaldson, S.L.; Comes, O.; Zuberi, K.; Badrawi, R.; Chao, P.; Franz, M.; Grouios, C.; Kazi, F.; Lopes, C.T.; *et al*. The GeneMANIA prediction server: Biological network integration for gene prioritization and predicting gene function. *Nucl. Acid. Res.* **2010**, *38*, doi:10.1093/nar/gkq537.

© 2013 by the authors; licensee MDPI, Basel, Switzerland. This article is an open access article distributed under the terms and conditions of the Creative Commons Attribution license (http://creativecommons.org/licenses/by/3.0/).
